# Sociodemographic factors associated with pregnant women's level of knowledge about oral health

**DOI:** 10.1590/S1679-45082018AO4079

**Published:** 2018-04-19

**Authors:** Wander Barbieri, Stela Verzinhasse Peres, Carla de Britto Pereira, João Peres, Maria da Luz Rosário de Sousa, Karine Laura Cortellazzi

**Affiliations:** 1 Hospital Israelita Albert Einstein, São Paulo, SP, Brazil.; 2 Núcleo de Estatística e Epidemiologia em Câncer, A.C. Camargo Câncer Center, São Paulo, SP, Brazil.; 3 Secretaria Municipal de Saúde, Coordenadoria Regional Sul, São Paulo, SP, Brazil.; 4 Faculdade de Odontologia de Piracicaba, Universidade Estadual de Campinas, Piracicaba, SP, Brazil.

**Keywords:** Pregnant women, Oral health, Prenatal care, Primary health care, Social conditions, Gestantes, Saúde bucal, Cuidado pré-natal, Atenção primária à saúde, Condições sociais

## Abstract

**Objective:**

To evaluate knowledge on oral health and associated sociodemographic factors in pregnant women.

**Methods:**

A cross-sectional study with a sample of 195 pregnant women seen at the Primary Care Unit Paraisópolis I, in São Paulo (SP), Brazil. For statistical analysis, χ^2^ or Fisher's exact test and multiple logistic regression were used. A significance level of 5% was used in all analyses.

**Results:**

Schooling level equal to or greater than 8 years and having one or two children were associated with an adequate knowledge about oral health.

**Conclusion:**

Oral health promotion strategies during prenatal care should take into account sociodemographic aspects.

## RESUMO

**Objetivo:**

Avaliar o conhecimento em saúde bucal e os fatores sociodemográficos associados em gestantes.

**Métodos:**

Estudo com delineamento transversal, com amostra de 195 gestantes atendidas na Unidade Básica de Saúde de Paraisópolis I, em São Paulo (SP). Para a análise estatística, utilizou-se teste χ^2^ ou teste exato de Fisher e a regressão logística múltipla. Assumiu-se um nível de significância de 5% para todas as análises.

**Resultados:**

Escolaridade igual ou maior a 8 anos de estudo e presença de um a dois filhos estiveram associadas a conhecimento adequado sobre saúde bucal.

**Conclusão:**

Estratégias de promoção de saúde bucal durante o pré-natal devem levar em consideração aspectos sociodemográficos.

## INTRODUCTION

The relation between social and health conditions of the populations has been investigated in the literature for some years.^(^ ^1^ ^-^ ^5^ ^)^ Acknowledging socioeconomic inequalities as determinants of increasing health inequities, as well as identifying the magnitude of these inequalities is essential for promotion of public policies that can reduce these differences.^(^ ^3^ ^)^ Some factors, such as level of schooling and perception of the need for treatment influence the attainment of knowledge and health care.^(^ ^6^ ^)^ Individuals with greater social deprivation have fatalistic beliefs about their health and less awareness of the need for care. In this context, health and wellbeing are concepts that express social and population beliefs, which, in turn, are influenced by cultural and demographic values resulting from how they relate to a territory and its characteristics.^(^ ^7^ ^)^ Social conditions exert a significant influence not only on the habits and behaviors of individuals, but also on their knowledge, perception and ability to self-manage health.

Oral health, as an integral part of human health, is also included in this context. Many studies investigating how social determinants affect health have permeated the scientific literature for a number of years, aiming to understand how oral diseases and habits relate with the social and economic conditions of the population.^(^ ^1^ ^,^ ^4^ ^,^ ^5^ ^,^ ^8^ ^-^ ^11^ ^)^ Oral health conditions derive from several factors, including poor income distribution, unemployment, low schooling and inadequate dental services. Economically disadvantaged people are disproportionately affected by dental caries and periodontal diseases. With regard to family income, scientific evidence shows that low family income is linked to poorer perception of one's oral condition, and the lower the income, the lower the proportion of people who have access to dental services.^(^ ^1^ ^,^ ^12^ ^)^

Among the phases of life in which social and demographic aspects can influence health conditions and even give rise to subgroups of greater vulnerability, we can highlight prenatal care. The literature considers pregnant women a strategic population group for the application of educational programs, recognizing pregnancy as a favorable phase for the establishment of healthier habits. Pregnant women are psychologically receptive to new knowledge, which makes them prone to adopting new and better health practices, the benefits of which extend to the rest of the family.^(^ ^7^ ^,^ ^10^ ^,^ ^11^ ^,^ ^13^ ^,^ ^14^ ^)^

Although programmatic actions in oral health have been well established and consolidated over the years, it is important to verify the knowledge future mothers have about oral health and how much this knowledge affects their oral health care practices.^(^ ^15^ ^)^ The incorporation of healthier behaviors by pregnant women depends on socioeconomic factors, such as education level, number of children, and age.^(^ ^6^ ^,^ ^16^ ^,^ ^17^ ^)^ In addition to the population's ingrained beliefs, low perception of one's need for oral care and a low level of knowledge about oral health lead to lower propensity to attain healthier habits and behaviors.^(^ ^7^ ^,^ ^18^ ^,^ ^19^ ^)^

Even when they wish to take proper care of their children's health, future mothers' unfavorable economic and social conditions make it difficult to practice what they learn.^(^ ^20^ ^)^

## OBJECTIVE

Evaluate pregnant women's knowledge on oral health and the associated sociodemographic factors.

## METHODS

A cross-sectional study, conducted at the Primary Care Unit (UBS – *Unidade Básica de Saúde*) Paraisópolis I in the southern region of the city of São Paulo (SP), in an area characterized by high levels of socioeconomic deprivation and a predominantly young population with many pregnant women, in the period from November 2011 to March 2012.

The convenience sample of pregnant women included all those living in the area covered by the UBS Paraisópolis I (n=228), and excluded those who were illiterate or had severe psychiatric disorders.

After confirmation of pregnancy, subjects were registered in the Prenatal Health Information System (SISPRENATAL - *Sistema de Informação em Saúde Pré-Natal)* and enrolled in two weekly group meetings organized by the Family Health Team.

At the first group meeting, a questionnaire adapted from Frazão et al., was applied by the oral health team to assess subjects' knowledge on oral health.^(^ ^15^ ^)^

We developed a score given by the sum of the answers to the questions, where each correct answer was equal to one point. This score was subtracted by the minimum value of the scale (zero), divided by the range of the scale (11-0), and then multiplied by 100. The score was divided into tertiles, classified as low (<37%), moderate (37-55%) and high knowledge (>55%). For data analysis, the category “low” meant inadequate knowledge, and the categories moderate and high were grouped as adequate knowledge.

Secondary demographic and socioeconomic data were collected from pre-natal records of the Mãe Paulistana program.

The dependent variable was oral health knowledge (dichotomized into adequate and inadequate). The independent variables were schooling (dichotomized by the median into <8 and ≥8 years of schooling), race (white, black, yellow and brown), work outside the home (no and yes), age group, number of children, previous pregnancies (no and yes) and abortions/miscarriages (no and yes).

First, a descriptive analysis of the data was carried out using absolute and relative frequencies, and measures of central tendency and dispersion. To investigate the association between independent variables and the dependent variable, the χ^2^ or Fisher's exact test was performed. Then, the variables with p<0.20 in the univariate analysis were tested in the multiple logistic regression model. A significance level of 5% was assumed for all analyses. The data were entered in Excel and analyzed using the Statistical Package of Social Science (SPSS), version 17.0, for Windows.

The study was approved by the Institutional Review Board of the City Health Department under # 319/11, CAAE: 0127.0.028.162-11.

## RESULTS

The final sample consisted of 195 pregnant women, with a mean age of 26.5 years and a standard deviation of 5.9.

According to table 1, 68.2% of pregnant women had over 8 years of schooling. The majority (83.9%) were white or brown. Only two pregnant women had high-risk pregnancies, and 46.2% were primigravidae. With regard to oral health knowledge, 74.4% of pregnant women had moderate to high level of knowledge.

**Table 1:** Sociodemographic characteristics and knowledge about oral health of pregnant women

Categorys		n (%)
Schooling, years		
	1-3	10 (5.1)
	4-7	52 (26.7)
	8-11	94 (48.2)
	≥12	39 (20.0)
Race		
	White	51 (26.4)
	Black	25 (13.0)
	Brown	113 (57.5)
	Yellow	6(3.1)
Work outside the home		
	No	97 (49.7)
	Yes	98 (50.3)
Work load/day*		
	4-8 hours	73 (75.3)
	9-14 hours	24 (24.7)
High-risk pregnancy		
	No	193 (99.0)
	Yes	2 (1.0)
Age, years		
	14-19	29 (14.9)
	20-29	112 (57.4)
	≥30	54 (27.7)
Children		
	None	90 (46.2)
	1-2	85 (43.6)
	≥3	20 (10.2)
Previous pregnancy		
	No	61 (31.3)
	Yes	134 (68.7)
Miscarriages/abortions		
	No	152 (77.9)
	Yes	43 (22.1)
Knowledge level		
	Low (<37%)	50 (25.6)
	Moderate (37-55%)	73 (37.5)
	High (>55%)	72 (36.9)

* Percentage over 98 pregnant women working outside home, showing an unknown value.

In table 2, regarding the first months of the baby's life, more than 60% of pregnant women correctly answered the questions about breastfeeding and how to clean the baby's mouth. Good hygiene and eating habits for a healthy dentition, the need for parental supervision for toothbrushing, and the use of fluoride in all phases of life were recognized as important by pregnant women.

**Table 2:** Mothers' knowledge about oral health

Questions		n (%)
As for redingtonite, it is correct to say that:		
	Suckling on the breast is an important exercise for developing the mouth and creating correct swallowing habits	11 (5.6)
	In the first six months of the baby's life, milk is essential and, ideally, breast milk because it has all nutrients needed for the baby and this her teeth formotion	42 (21.5)
	Breast milk has antibodies responsible for the body's defense	7 (3.6)
	Breastfeeding is a source of satisfaction, pleasure and safety to the child.	5 (2.6)
	All alternatives are correct*	118 (60.5)
	I don't know	12 (6.2)
Regarding oral hygiene of the newborn, which statement is correct:		
	Use toothbrush only	5 (2.6)
	Use toothbrush and fluoride toothpaste	8 (4.1)
	Use gauze or cloth wet with clean water*	130 (66.7)
	Since babies do not have teeth, it is not necessary to clean their mouth	16 (8.2)
	Only the dentist should clean the baby's mouth	7 (3.6)
	I don't know	29 (14.9)
In your perception, which statement is correct in respect to baby teeth:		
	Since they are temporary, baby teeth do not need any sort of care	8 (4.1)
	They guide the eruption or “coming in” of permanent teeth*	67 (34.4)
	They emerge in the mouth when the mother stops breastfeeding the baby	5 (2.6)
	They fall easily because they have no roots	41 (21.0)
	I don't know	74 (37.9)
Regarding children's oral hygiene, which statement is correct:		
	From 2 years of age, children can brush their own teeth	10 (5.1)
	Children start flossing from the age of 7	8 (4.1)
	Parents should help and supervise brushing and flossing of their children up to age 6*	154 (79.0)
	Up to age 3, children must not use toothpaste	6 (3.1)
	I don't know	17 (8.7)
At what age does the first permanent tooth start to emerge?		
	Around 6 months to 1 year old	38 (19.5)
	From 2 to 3 years old	19 (9.7)
	From 5 to 6 years old*	61 (31.3)
	From 8 to 9 years old	32 (16.4)
	From 11 to 12 years old	8 (4.1)
	I don't know	37 (19.0)
Some people have strong teeth due to:		
	Transmission from parents (heredity)	8 (4.1)
	Types of races	3 (1.5)
	Good financial condition	2 (1.0)
	Good oral hygiene and eating habits*	170 (87.2)
	I don't know	12 (6.2)
Dental caries is not primarily caused by:		
	Malformation of the tooth structure	41 (21.0)
	Bacteria on teeth	6(3.1)
	Constant use of antibiotics*	25 (12.8)
	Lack of saliva in the mouth	24 (12.3)
	Frequent intake of sugary products	18 (9.2)
	I don't know	81 (41.5)
To avoid inflammation of the gums, it is necessary to perform oral hygiene procedures, correctly using:		
	Toothbrush only	4 (2.1)
	Toothbrush and fluoride toothpaste only	37 (19.0)
	Toothbrush and floss*	81 (41.5)
	Special mouthwashes and fluoride solutions	53 (27.2)
	I don't know	20 (10.3)
During pregnancy, the increase in deantal caries occurrences is due to:		
	Weakening of teeth by loss of calcium	57 (29.2)
	Higher intake of sugary products*	39 (20.0)
	Hormonal changes	37 (19.0)
	Drugs (e.g. antibiotics)	13 (6.7)
	I don't know	49 (25.1)
During pregnancy, do you think dental treatment should be:		
	Preventive and regular*	118 (60.5)
	Avoided during the entire pregnancy period	18 (9.2)
	In cases of urgency	19 (9.7)
	For pregnant women who do not use fluoridated water	3 (1.5)
	I don't know	37 (19.0)
Fluoride is important:		
	Only in childhood, at the time of teeth formation and eruption	2 (1.0)
	In adult life	9 (4.6)
	In all phases of life*	162 (83.1)
	I don't know	22 (11.3)

* This statement is the right answer

Most of the women had doubts about the etiology of dental caries disease and only 20% of the sample identified changes in eating habits during pregnancy as a risk factor. In addition, 30% of sample recognized the importance of deciduous teeth and transitional dentition for oral health. Concerning the prevention of gum disease, only 41.5% reported using toothbrush and floss to prevent gingivitis. Regarding dental care during pregnancy, 60.5% think it should be regular and preventive.

Table 3 shows that inadequate knowledge about oral health was lower in pregnant women with ≥8 years of schooling odds ratio (OR) (OR=0.48, p=0.032). The age group of 30 and over was a protective factor (OR=0.28; p=0.020) for inadequate knowledge. Having between one and two children was a protective factor for inadequate oral health knowledge (OR=0.30, p=0.001), and women with children had a lower chance of having inadequate knowledge when compared to pregnant women without children (OR=0.37, p=0.004).

**Table 3:** Association of independent and dependent variables by χ^2^ or Fisher's exact test

Adequate		Knowledge about oral health		χ^2^	OR	95%CI	p value
		Adequate n (%)	Inadequate n (%)				
Scholling, years							
	1-7	40 (64.5)	22 (35.5)	0.032	1.0		
	≥8	105 (78.9)	28 (21.1)		0.48	0.25-0.94	0.033
Race							
	White	41 (80.4)	10 (19.6)	0.269	1.0		
	Black, brown, yellow	103 (72.5)	39 (27.5)		1.55	0.71-3.40	0.271
Work outside home							
	No	67 (69.1)	30 (30.9)	0.093	1.0		
	Yes	78 (79.6)	20 (20.4)		0.57	0.30-1.10	0.094
Work load/day, hours							
	4-8	58 (79.5)	15 (20.5)	1.000	1.0		
	9-14	19 (79.2)	5 (20.8)		1.02	0.33-3.17	0.976
Age range, years							
	14-19	18 (62.1)	11 (37.9)	0.053	1.0		
	20-29	81 (72.3)	31 (27.7)		0.63	0.27-1.47	0.284
	≥30	46 (85.2)	8 (14.8)		0.28	0.10-0.82	0.020
Children							
	None	56 (62.2)	34 (37.8)	0.002	1.0		
	1-2	72 (84.7)	13 (15.3)		0.30	0.14-0.62	0.001
	≥3	17 (85.0)	3 (15.0)		0.29	0.08-1.10	0.062
Previous pregnancy							
	No	37 (60.7)	24 (39.3)	0.003	1.0		
	Yes	108 (80.6)	26 (19.4)		0.37	0.19-0.72	0.004
Miscarriages/abortions							
	No	114 (75.0)	38 (25.0)	0.700	1.0		
	Yes	31 (72.1)	12 (27.9)		1.16	0.54-2.48	0.700

The category “inadequate” refers to a dependent variable. χ^2^ or Fisher's exact test. OR: *odds ratio*; 95%CI: 95% confidence interval.

In the multiple logistic regression analysis (Table 4), schooling and number of children were independent protective factors for inadequate oral health knowledge. Pregnant women with ≥8 years of schooling had a greater chance of having adequate knowledge, and having one or two children was a protective factor for inadequate knowledge (OR=0.34, p=0.007) when compared to women without children.

**Table 4:** Multiple logistic regression analysis

Variables		Adjusted OR*	95%CI	p value
Schooling, years				
	1-7	1.0		
	≥8	0.36	0.17-0.75	0.007
	None	1.0		
Children				
	1-2	0.34	0.16-0.75	0.007
	≥3	0.36	0.07-1.76	0.208

* OR adjusted by the continuous variable age of pregnant woman; the category “inadequate” refers to a dependent variable.

OR: odds ratio; 95%CI: 95% confidence interval.

## DISCUSSION

Although most of the pregnant women showed moderate to high levels of knowledge, there are knowledge gaps in the study group concerning some areas of oral health.

In this study, most of the respondents acknowledged the importance of breastfeeding and its benefits, a result similar to that of Simioni et al., where all pregnant women interviewed intended to breastfeed their children and believed breastmilk was the best food for the baby in their first year of life.^(^ ^21^ ^)^

Breastfeeding is considered the most natural and important way to physiologically, physically and psychologically nurture an infant. The health and nutritional status of mothers and children are closely linked, and promoting breastfeeding is an important strategy to encourage healthy infant feeding practices.^(^ ^10^ ^)^

Even with good understanding of oral hygiene of the newborn and awareness of the need for parental supervision in children's oral health, there is still misinformation among pregnant women about the importance of deciduous teeth and the age at which the first permanent teeth emerge. Frazão et al., showed similar results, however his study was not restricted to pregnant women.^(^ ^15^ ^)^ Early loss of deciduous teeth can have serious consequences for permanent dentition, increasing the likelihood of orthodontic problems, feeding difficulties and even psychological impairment of the child.^(^ ^22^ ^)^

In this study, only 20% of pregnant women associated dental caries with changes in eating patterns during pregnancy, and most shifted the issue to other factors, such as tooth weakening due to calcium loss (29.2%), hormonal changes (19%), and use of drugs (6.7%). In the study by Simioni et al., 48.75% of pregnant women said dental caries in pregnancy was normal, due to the transfer of calcium from the mother's teeth to baby's teeth.^(^ ^21^ ^)^ The notion of a relation between pregnancy and dental problems is grounded on popular values and beliefs. However, it is known that dental caries is not directly associated with pregnancy, but rather to an increase in eating frequency, coupled with careless oral hygiene during this phase.^(^ ^19^ ^,^ ^23^ ^,^ ^24^ ^)^ Also added on top is the limited knowledge pregnant women have about oral hygiene.^(^ ^25^ ^-^ ^28^ ^)^

Most pregnant women (58.5%) in this study did not identify brushing and flossing as the most effective ways to prevent gum disease. The use of dental floss may be conditioned to the socioeconomic situation, since this is a relatively expensive product, inaccessible to a significant portion of the population. Similar results were found by Chung et al., Bamanikar et al., and Melo et al., regarding daily flossing to prevent oral diseases (40.9%, 42%, and 44%, respectively, reported using the method).^(^ ^8^ ^,^ ^14^ ^,^ ^29^ ^)^ Bastiani et al., revealed that only 20% of pregnant women identified brushing their teeth as a way to prevent gingivitis.^(^ ^30^ ^)^ Frazão et al., observed that the primary method to prevent gingivitis, according to respondents, was the use of mouthwashes.^(^ ^15^ ^)^ According to the authors, this could be due to media campaigns encouraging the use of antiseptics for bad breath and bacterial plaque control. The study by Gouvêa et al., who looked into community health agents' knowledge about oral health, showed that these professionals, when integrating an oral health team, had excellent knowledge about the evolution of gingival bleeding and the preventive measures to improve this condition.^(^ ^22^ ^)^

Regarding the perception of what determines “strong” teeth, the pregnant women in this study pointed to with the importance of oral hygiene and diet, rather than alternatives such as heredity, race and financial condition, corroborating the results of Frazão et al.^(^ ^15^ ^)^

As for dental treatment during pregnancy, most of the respondents (60.5%) said it should be preventive and regular. However, there is still a strong presence of beliefs relative to dental care during pregnancy, both in the general population and among health professionals.^(^ ^20^ ^)^ There are deep-rooted myths and barriers about dental care during pregnancy pointing to concerns about the possibility of detrimental effects to the baby's health.^(^ ^18^ ^)^ Misinformation of pregnant women about the importance of taking care of their oral health is one of the reasons they do not seek dental care. Other barriers reported by pregnant women were the waiting time to attain dental care, distance from home to the health facility, and negative attitudes of health professionals.^(^ ^14^ ^)^ Dental care should be a part of prenatal care, with better integration of health professionals, and standardization of the guidance provided on the importance of dental care during pregnancy.

This study concluded that 8 or more years of schooling and having one to two children were associated with an adequate level of knowledge about oral health in pregnant women. These associations can be explained by the fact that underprivileged parents are less capable of taking care of themselves and their children. Worse social determinants are associated with less access to information and a lower level of education.^(^ ^9^ ^)^

The consequences of social deprivation among pregnant women in the community investigated have been tempered by the increased access to dental services, particularly since 2008, with the establishment of oral health teams at the UBS Paraisópolis I, incorporating dental care into the work of Family Health Teams in this unit. During this period, the access of pregnant women to preventive actions in oral health increased significantly.

Education level is an important marker of socioeconomic status, and high levels of schooling are usually predictors of better working and housing conditions.^(^ ^4^ ^)^ Families with higher education levels are likely to present more positive attitudes and knowledge about preventive oral care.^(^ ^2^ ^,^ ^14^ ^)^ Studies show that mothers with higher education levels positively influence the oral health of their children.^(^ ^16^ ^)^ Low education levels are associated with low oral health literacy.^(^ ^11^ ^)^

In the study conducted by Bamanikar et al., investigating the oral health knowledge and practices of 95 pregnant women, the authors concluded that the pregnant women's knowledge of oral health is associated with their education level and working condition.^(^ ^14^ ^)^ Nogueira et al., observed a correlation between the number of children and the level of schooling, indicating that the lower the schooling, the greater the number of children.^(^ ^7^ ^)^

Still on the number of children, this study observed that mothers with one or two children were less likely to have inadequate oral health knowledge than mothers without children. We can also attribute these results to repeated exposure to oral health information in multiple past situations, such as educational groups and consultations during prenatal care at the primary care unit, since dental follow-up in pregnant women has always been a priority of the programmatic oral health actions under the Family Health Strategy. Access to dental services can therefore be considered a positive factor leading to the majority of pregnant women in the Paraisópolis community having an adequate level of knowledge about oral health.

Past experience from a previous pregnancy can help direct prenatal oral health interventions. Primigravidae, despite their inadequate knowledge about oral health, may be considered prone to incorporating new habits, since future mothers are likely to be more open to acquiring knowledge and learning new health-related practices.^(^ ^17^ ^)^

A possible limitation of this study was the fact that this was a convenience sample restricted to the study site, making it difficult to generalize the findings.

## CONCLUSION

The majority of pregnant women showed an adequate level of knowledge about oral health, but there are some gaps regarding aspects of oral care, particularly preventive methods, etiology of dental caries and myths about dental treatment during pregnancy. Pregnant women with higher educational level and who had one to two children had a lower chance of having insufficient knowledge about oral health.
